# Effect of eggplant (*Solanum melongena*) on the metabolic syndrome: A review

**DOI:** 10.22038/ijbms.2021.50276.11452

**Published:** 2021-04

**Authors:** Fatemeh Yarmohammadi, Mahboobeh Ghasemzadeh Rahbardar, Hossein Hosseinzadeh

**Affiliations:** 1 Student Research Committee, Mashhad University of Medical Sciences, Mashhad, Iran; 2 Department of Pharmacodynamics and Toxicology, School of Pharmacy, Mashhad University of Medical Sciences, Mashhad, Iran; 3 Pharmaceutical Research Center, Pharmaceutical Technology Institute, Mashhad University of Medical Sciences, Mashhad, Iran

**Keywords:** Antihypertensive, Antihyperlipidemic, Aubergine, Diabetes, Eggplant, Metabolic syndrome, Solanum melongena

## Abstract

Metabolic syndrome (MetS), also known as syndrome X, is a significant risk factor for cardiovascular disease incidence and mortality. Increasing age, obesity, physical inactivity, smoking, and positive family history are the risk factors associated with MetS, which increases the risk of diabetes, cardiovascular disease, hypertension, hyperlipidemia, and obesity. Chemical compounds in the treatment of metabolic complications are associated with a lack of efficacy and severe side effects. Numerous studies have described the importance of herbs and natural products to treat human diseases. Therefore, nowadays, herbs-based diets and herbal medicines are recommended for the management of various diseases. The protective effects of several herbs have been reported against MetS such as rosemary, avocado, and silymarin. Eggplant (*Solanum melongena*) is a rich source of phenolic and alkaloid compounds. It possesses various pharmacological effects, including, anti-oxidant, antidiabetic, antihypertensive, and antihyperlipidemic, which has been supported by numerous investigations. In this review, we evaluated the effects of eggplant on MetS and its complications comprising diabetes, high blood pressure, hyperlipidemia, and obesity.

According to these studies, eggplant can control diabetes through the anti-oxidative properties and inhibition of α-amylase and α-glucosidase activity. Also, eggplant has exerted an antihypertensive effect via ACE inhibitory activity. Eggplant may have shown protective effects on hyperlipidemia and obesity via the induction of lipoprotein lipase activity and the reduction of pancreatic lipase activity.Eggplant can be useful in the treatment of MetS and its complications.

## Introduction

Metabolic syndrome (MetS), also known as syndrome X, is not a disease in itself. Instead, patients with metabolic syndrome often develop diabetes, abdominal obesity, hyperlipidemia, and hypertension (1-3). MetS prevalence is the highest among the population over 60 years, and it is increasing among adolescents and children (4). The risk of cardiovascular disease will increase when MetS occurs (5, 6). Since the MetS has a high rate of mortality, thereby, lifestyle modification (calorie restriction, physical activity, and adequate sleep) and pharmacological therapy are essential (7, 8). Several studies have been conducted to assess the impact of medicinal plants or their extracts against MetS and its complications. For instance, in a review study published in 2017, it was concluded that mangosteen might help improve MetS and the quality of life (9). Moreover, rosemary (10), silymarin (11), avocado (12), cinnamon (13), and *Abelmoschus* *esculentus* (14) are herbs with protective effects against MetS. 

**Figure 1:**
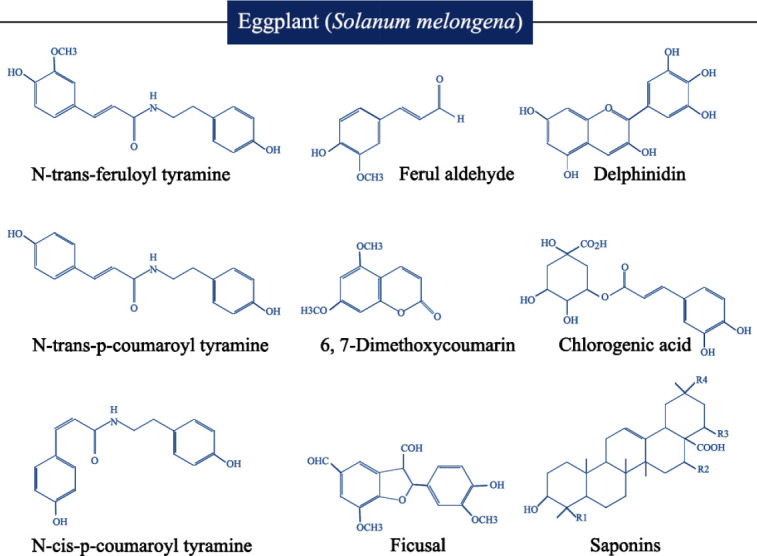
The structure of the main compounds in the skin, pulp, and root of eggplant

Eggplant (*Solanum* *melongena*) is one of the common plants grow all around the world, especially in Asian countries, the Middle East, and around the Mediterranean basin (15, 16). Eggplant belongs to the Solanaceae family and the genus Solanum (17). Based on the fruit shape, eggplant has been classified into three types including, egg-shaped, long slender shape, and dwarf types (18). It also has known as aubergine and it is an important source of fiber, minerals (iron, calcium, potassium, magnesium, sodium, zinc, and phosphorus), vitamins C, thiamin, niacin, B6, B12, A, E, D, and K (19). Eggplant has been used in the treatment of several diseases, including asthma, bronchitis, diabetes, arthritis, and hypercholesterolemia (20, 21). The clinical use of eggplant is due to its phenolic and alkaloid contents (16). The delphinidin (an anthocyanin) and chlorogenic acid (a phenolic acid) are the main phenolic compounds in the skin and pulp of eggplant (18) (Figure 1). 

It has been reported that delphinidin can induce endothelial vasodilation by the activation of the nitric oxide (NO) pathway (22). Delphinidin also has shown antihypertensive effects via interrupting the renin (an aspartyl protease)-angiotensin system (RAS) signaling pathway (23). The overactivation of this system is one of the most important risk factors for the development of hypertension (24). Delphinidin reduces the expression and activation of the angiotensin-converting enzyme (ACE) (23). *α*-Amylase, as an enzyme in saliva and pancreas, plays an essential role in carbohydrate digestion. The inhibition of salivary and pancreatic α-amylase enzymes can reduce glucose uptake (25). Moreover, delphinidin has exhibited inhibitory properties against the α-amylase enzyme, and thereby it may be useful in the treatment of diabetes and its complications such as overweight and obesity, and cardiovascular disease (26). 

Chlorogenic acid has been found to enhance NO status, improve endothelial function, and lower blood pressure (27). Also, chlorogenic acid has shown beneficial effects on type 2 diabetes mellitus by increasing the translocation of glucose transporter 4 (GLUT4) to the plasma membrane, enhancing glucose transport to skeletal muscle, and inhibiting gluconeogenesis, besides delaying intestinal glucose absorption (28). It has been suggested that chlorogenic acids exhibit anti-obesity and antihyperlipidemic activities by alleviating the levels of free fatty acids and triglycerides (TG) (29). Additionally, several studies have reported the pharmacologic aspects of eggplant, such as anti-oxidant (30), anti-inflammatory (31), antibacterial (32), antifungal (33), antidiabetic (34), antihypertensive (35), anti-obesity (36), hepatoprotective (37), and hypolipidemic (38) properties. Thus, the purpose of this study is to review the effects of eggplant on MetS.

In this review, various databases such as PubMed, Scopus, and Google Scholar have been involved. All the articles that have been chosen in this review were collected from the date of inception up to May 2020. The search keywords contained “eggplant”, “*Solanum* *melongena*”, “aubergine”, “metabolic syndrome”, “diabetes”, “hyperglycemia”, “insulin”, “hypoglycemic”, “antihyperglycemic”, “antidiabetic”, “blood glucose”, “hypertension”, “blood pressure”, “hypotensive”, “antihypertensive”, “dyslipidemia”, “hyperlipidemia”, “high cholesterol”, “high triglyceride”, “hypercholesterolemia”, “hypertriglyceridemia”, “atherogenic”, “atherosclerosis”, “obesity”, “overweight”, “appetite”, “anti-obesity” and “weight loss”.

***Effects of eggplant on diabetes***

Diabetes is a common metabolic disorder identified by abnormally high blood glucose levels and the insufficiency of secretion or function of endogenous insulin (39, 40). It is leading to major complications, such as diabetic retinopathy (41), neuropathy (42), nephropathy (43), micro-angiopathy (44), and cardiovascular diseases (45). It has been reported that several natural compounds such as green tea and garlic have improved health status in diabetes patients (46, 47). A study has investigated the efficacy of methanol extract of African eggplant leaves (100, 200, and 300 mg/kg, orally for 20 days) on alloxan-induced diabetes in rats. The results of this study showed that eggplant reduces high blood glucose levels in diabetic rats (48). The main mechanisms in the pathogenesis and progression of diabetes are free radicals production and oxidative stress. Oxidative stress can increase the development of diabetes-related complications including cardiovascular disease, neuropathy, and kidney disease (49). A significant protective pathway against oxidative stress is the activation of the nuclear erythroid 2-related factor 2 (Nrf2)/ anti-oxidant response element (ARE) signaling pathway. Nrf2 is a transcription factor that binds to the ARE and thereby up-regulates the anti-oxidant gene expression such as superoxide dismutase (SOD) and heme oxygenase-1 (HO-1) (50). The protein kinase C (PKC) and Kelch-like ECH-associated protein 1 (Keap1) are intracellular redox sensors. Under basal conditions, Keap1 inhibits the Nrf2/ARE signaling pathway through direct interaction with Nrf2. Under oxidative stress conditions, elevated ROS interacts with cysteine residues of Keap1 and dissociate it from Nrf2. ROS also regulate PKC activity and result in the phosphorylation and activation of Nrf2. Then Nrf2 translocates from the cytoplasm into the nucleus and induces the expression of the anti-oxidant enzyme genes such as SOD, heme oxygenase-1 (HO-1), and NAD (P) H quinone oxidoreductase 1 (NOQ1) (51). The imbalance between ROS production and anti-oxidant enzyme expression leads to β-cell dysfunction and insulin resistance (51, 52). Therefore, the Nrf2 signaling pathway is able to act as a potential therapeutic target in diabetes. Another study has reported that purple eggplant contains anthocyanin compounds that exerted anti-oxidant properties. The effect of the aqueous extract of purple eggplant (343, 686, and 1372 mg/kg, orally, for 14 days) has investigated on alloxan-induced diabetes (150 mg/kg, IP) in rats. The results of this study have shown that the extract of eggplant (686 and 1372 mg/kg) reduced the level of serum malondialdehyde (MDA) in diabetic rats (53). Several enzymes are involved in the absorption of glucose in the digestive organs, such as α-glucosidase (α-d-glucoside glucohydrolase) and α-amylase (α-1, 4-glucan-4- glucanohydrolases). α-glucosidase acts as the key enzyme in catalyzing the final level of carbohydrates digestion. Inhibiting these enzymes can delay glucose absorption and slows the elevation of blood glucose levels (54). 

Numerous studies have reported that different phytochemicals can inhibit α-glucosidase activity (55-57). The phenolic, glycoalkaloids, and carotenoids compounds are found abundantly in eggplants (19). α-Amylase is secreted by the pancreas and salivary glands and plays an essential role in the digestion of starch and glycogen (58). Eggplant is an important source of flavonoids and has the potential to inhibit α-amylase activity (58, 59). A study has compared the total soluble phenolic content of the pulp and skin of eggplant varieties, including Purple, White, Graffiti, and Italian eggplants. This study has reported that the skin aqueous extract of Italian eggplant has the highest soluble phenolic content. Moreover, the results of this study exhibited that the skin of Italian eggplant had the highest anti-oxidant activity, followed by the pulp extract of White, Graffiti, and Purple. Also, the pulp and skin extracts of eggplant have shown an inhibitory effect on the intestinal α-glucosidase, except the Italian variety. Additionally, this study suggests that the eggplant varieties were moderate α-amylase inhibitors, except for Graffiti pulp (60). Therefore, eggplant has the potential to reduce glucose absorption in the intestine. An *in* *vitro* study has investigated the inhibitory effects of two eggplant species (*S.* *melongena* and *S.* *macrocarpon*) on starch hydrolyzing enzymes. The results of this study showed that the phenolic content of *S. macrocarpon* is higher than *S. melongena* extract and the methanolic extracts of both species inhibited α-glucosidase activity more than α-amylase activity in a dose-dependent manner (59). In another assessment, six phenolic compounds of the root of *S.* *melongena *were isolated (*N-trans*-feruloyl tyramine, *N-trans-p-*coumaroyl tyramine, *N-cis-p*-coumaroyl tyramine, Ferul aldehyde, 6, 7-Dimethoxycoumarin, and Ficusal) (Figure 1) and the inhibitory effect of these compounds against α-glucosidase was evaluated. The results of this study have shown that *N-trans*-feruloyl tyramine, *N-trans-p-*coumaroyl tyramine, and *N-cis-p*-coumaroyl tyramine have the highest inhibitory impact, besides Ferul aldehyde, 6,7-Dimethoxycoumarin, and Ficusal did not show any inhibitory activity (61).

**Figure 2:**
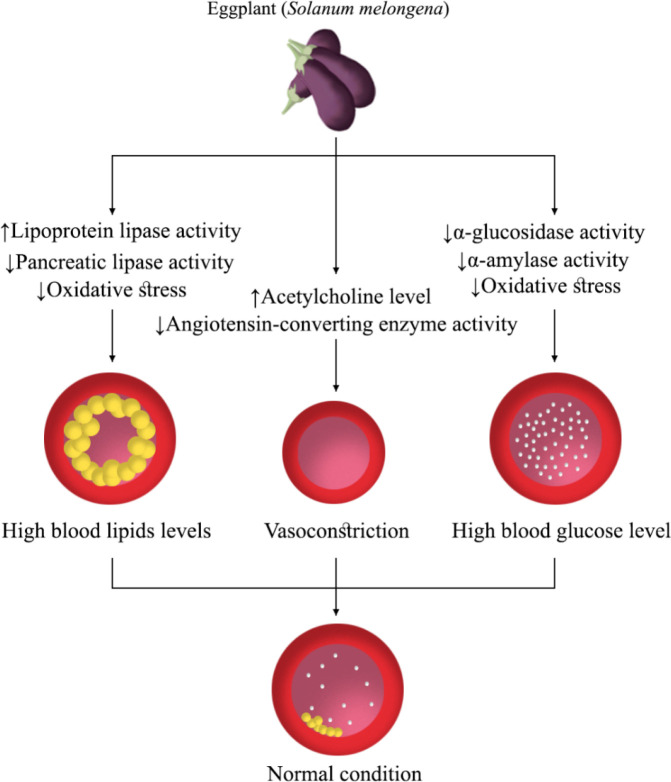
Main mechanisms of eggplant on high blood pressure, high blood glucose, and lipid levels

Generally, these studies revealed that eggplant could manage/prevent diabetes and its complications through the anti-oxidative properties and inhibition of α-amylase and α-glucosidase activity (Figure 2).

***Effect of eggplant on high blood pressure***

High blood pressure (BP), a common chronic condition, is created by the high force of the blood against artery walls and vascular dysfunction, which indirectly induces the risk of cardiovascular disease and type 2 diabetes (62, 63). The RAS plays a vital role in the regulation of body fluid volume and BP. Under physiological conditions, kidneys release renin into the circulation and convert angiotensinogen which is released from the liver to angiotensin I. Then, angiotensin I is converted to angiotensin II via the action of ACE, which bonds to the endothelium. Angiotensin II acts as a potent vasoconstrictor and regulates BP. Also, ACE can metabolize bradykinin (a vasodilator) and regulates BP. Therefore, the blockade of the RAS by ACE inhibitors is effective in managing high BP (64). To date, several natural compounds are identified to be effective in the treatment of high BP, such as rosemary and saffron (10, 65). A document compared ACE inhibitory activity of the pulp and skin of eggplant varieties, including Purple, White, Graffiti, and Italian eggplants. The pulp extract of White showed the highest ACE inhibitory activity in a dose-dependent manner, followed by the skin extract of White and pulp extract of Graffiti. The inhibition of this enzyme provides a robust biochemical basis for the management of high BP (60). Acetylcholine (ACh) is an endothelial-dependent vasodilator, which plays a significant role in the reduction of the pathogenesis of hypertension. Following the stimulation of muscarinic receptors located on the vascular endothelium by ACh, nitric oxide (NO) is released. NO relaxes arterial smooth muscle and causes a fall in arterial blood pressure (66).

*S.* *melongena* contains abundant ACh and can exert an antihypertensive effect. Also, it has been reported that oral administration of *S.* *melongena* (0.0650 and 0.821 mg/kg, for 28 days) reduced systolic blood pressure (SBP) and diastolic blood pressure (DBP) levels by ACh in rat thoracic aorta rings (67). The sympathetic nervous system (SNS) and the adrenal medulla play a significant role in BP regulation via the release of epinephrine and norepinephrine (68, 69). On the other hand, the excessive release of epinephrine and norepinephrine results in increased vascular resistance, cardiac contractility, and cardiac output (70). A study on the effect of aqueous extract of eggplant (0.001 to 1 mg/ml) on norepinephrine-stimulated guinea pig atria has shown that eggplant decreased the high BP probably via decreasing the cardiac output and myocardial contractility (71).

In these studies, ACh has been introduced as the main compound responsible for the antihypertensive effect of eggplant. Also, eggplant has exerted an antihypertensive effect via ACE inhibitory activity. Therefore, eggplant has been suggested as useful food for the management of hypertension and its complications in daily life (Figure 2).

***Effect of eggplant on hyperlipidemia***

Hyperlipidemia is abnormally elevated levels of lipids, including fat, cholesterol, and TGs in the blood, which is commonly known as the main risk factor for cardiovascular disease and is one of the leading causes of mortality worldwide (72, 73). Eggplant is rich in dietary fiber and possibility can positively modify the lipid profile, including total cholesterol (TC), low-density lipoprotein cholesterol (LDL-C), high-density lipoprotein cholesterol (HDL-C), very low-density lipoprotein cholesterol (VLDL-C), total triglyceride (TG), in addition to apolipoprotein (Apo) A and B (74). In a clinical study, the influence of eggplant on lipid profile (TC, TC, LDL-C, HDL-C, and TG) was examined in 19 healthy subjects with a mean age of 26 years (11 females and 8 males) for three weeks. The obtained results revealed that 200 ml orange juice with fresh unpeeled eggplant did not modify lipid profiles in the study population (75). In another research, the effects of ingesting eggplant infusion on TC and TG in the serum of 38 hypercholesterolemic patients with the mean age of 43.7 years were assessed for five weeks. The results of this study displayed that eggplant has a modest and transitory effect on the parameters analyzed, including TC, LDL-C, HDL-C, VLDL-C, TG, Apo A, and Apo B (21). Furthermore, the effect of eggplant on serum lipid profile with lovastatin was compared in 21 individuals (both sexes) with TC levels of more than 200 mg/dl. In this study, eggplant did not show a significant effect on TC, LDL-C, and HDL-C levels after six weeks. Therefore, eggplant cannot be considered as an alternative to lovastatin in decreasing cholesterol serum levels (76). Additionally, the effects of eggplant (capsules 450 mg of dried powdered fruits, twice daily, for three months) were evaluated on 41 hyperlipidemic patients. The data revealed that eggplant decreased the serum levels of TC and LDL-C (77). 

In an animal study, the influence of eggplant on cholesterol metabolism in rats fed with diets containing 1 % eggplant has been reported. The results illustrated that the leaf and fruit of eggplant could not lower the cholesterol pool of the serum and liver in rats. However, it was reported that eggplant reduced the absorption of a single dietary dose of cholesterol, probably via binding the bile salts to cholesterol (78). Besides, the effect of eggplant (free access to extract, orally, for 12 weeks) on cholesterol metabolism and atherosclerosis has been studied in LDL receptor knockout mice. In this study, eggplant did not decrease total cholesterol and atherogenic lipoprotein levels (79). In another study, the hypolipidemic effect of the isolated flavonoids from eggplant (dose of 1 mg/100 g BW, orally) was tested in cholesterol-fed rats. It was observed that eggplant stimulated lipoprotein lipase activity, and attenuated the concentration of TG in serum. Moreover, eggplant decreased serum cholesterol concentration and triggered 3-hydroxy-3-methylglutaryl-coenzyme A (HMG-CoA) reductase activity (80). HMG-CoA reductase is an enzyme in the liver that catalyzes the formation of cholesterol from HMG-CoA (81). Therefore, it might be suggested that eggplant reduced cholesterol levels via some other mechanisms, including the enhancement rate of degradative processes of elimination of cholesterol and the effective reduction in its absorption from the intestine (82). Also, the effects of eggplant (a diet containing 61.62%, orally, for 28 days) on serum and hepatic TC and TG have been investigated in cholesterol-fed rats. It was indicated that eggplant aqueous extract decreased hepatic TC, while it had little effect on serum and hepatic TG (83). In rats treated by the high-fat diet, assessing the effect of eggplant peels powder (a diet containing 4 % eggplant for four weeks) on lipid profiles revealed that eggplant modified TC, LDL-C, HDL-C, and VLDL-C levels (84). Administration of methanol extract of African eggplant leaves (100, 200, and 300 mg/kg, orally) to diabetic rats resulted in a significant reduction in the TC, LDL-C, HDL-C, and TG values (48). Also, the eggplant core reduced the plasma concentration of TC, LDL-C, and HDL-C in guinea pigs fed a hypercholesterolemic diet (85). In an experiment in hypercholesterolemic rabbits, the treatment of 10 ml eggplant juice daily for 2 weeks significantly reduced weight and plasma cholesterol levels (86). 

Generally, in these studies, the possible mechanisms of action of eggplant on cholesterol metabolism and lipid profile have not been clarified. Since the eggplant has induced HMG-CoA reductase activity, eggplant may have shown protective effects on hyperlipidemia via the enhancement rate of degradative processes of cholesterol or induction of lipoprotein lipase activity, and the effective reduction in lipids absorption from the intestine (Figure 2). Although experimental animal studies have demonstrated the hypolipidemia effect of eggplant, clinical studies suggested a small transient effect of eggplant on hyperlipidemia, which might be due to the small number of individuals in studies and the use of low eggplant doses to induce hypolipidemic effects.

***Effect of eggplant on obesity***

Obesity is a medical problem in which excess body fat has accumulated to the extent that it may harm health and is defined by body mass index (BMI), an indicator of the measure of high body fatness (87, 88). It acts as a risk factor for several diseases such as type 2 diabetes (89), hypertension (90), cardiovascular (91), and respiratory diseases (92). The anti-oxidant status and ROS formation are involved in metabolic disorders associated with obesity (93). Several studies have shown that the plasma levels of anti-oxidant markers (SOD, GSH) are decreased in obese patients (93-95). The anti-oxidant compounds are used in the treatment of obesity and its complications (96, 97). The pancreatic lipase acts as a key enzyme in intestinal fat digestion. Therefore, pancreatic lipase inhibitors such as saponins and phenolic compounds might be introduced as therapeutic targets in controlling obesity (98-100). An *in* *vitro* study investigated the inhibitory effect of eggplant on pancreatic lipase activity. The saponins isolated from the methanol extract of eggplant have shown an inhibitory effect on porcine pancreatic lipase activity (the titrimetric method) (Figure 1) (101). 

A randomized clinical trial has reported the effects of eggplant flour (13 g, orally) on anti-oxidant status and body fat in 97 overweight women (the mean age of 47.5 years) for four months. This study has shown that eggplant increased the anti-oxidant capacity in plasma and reduced the body fat mass in volunteers (36).

Because eggplant has the potential to reduce pancreatic lipase activity (Figure 2), future studies can investigate the anti-obesity effects of eggplant. 

**Table 1:** The protective effects of eggplant on metabolic syndrome

Pharmacological effects	Compound	Study design	Results	Ref
Anti-diabetic	Leaves, 100, 200, and 300 mg/kg, orally, for 20 days	Rats, alloxan	↓BG	(48)
	Aqueous extract of purple eggplant, 343, 686, and 1372 mg/kg, orally, for 14 days	Rats, alloxan	↓BG	(53)
	Skin and pulp aqueous extracts	*In vitro*	↓Oxidative stress ↓α-glucosidase activity	(60)
	Methanolic extracts	*In vitro*	↓α-glucosidase activity ↓α-amylase activity	(59)
	Root ethanolic extract	*In vitro*	↓α-glucosidase activity	(61)
Anti-hypertensive	Skin and pulp aqueous extracts	*In vitro*	↓ACE activity	(60)
	Calyx, 0.0650 and 0.821 mg/kg, orally	Spontaneously hypertensive rats	↓BP	(67)
	Unripe fruits aqueous extracts	Guinea pig atria, norepinephrine	↓BP	
Anti-hyperlipidemic	2% (w/v) infusion	Human (11 men and 27 women), a RCT	↓TC and LDL-C levels ↓Apo B	(21)
	The eggplant and orange blended juice	Human (8 men and 11 women), a RCT	Lipid profile was not modified	(75)
	_	Human (9 men and 12 women)	Lipid profile was not modified	(76)
	Capsules 450 mg	Human (41 women), a double-blind placebo-controlled study	↓TC and LDL-C levels	(77)
	Leaf or fruit powder	Rats	Lipid profile was not modified ↓Absorption of cholesterol	(78)
	Fruit aqueous extract	LDL receptor knock out mice	Lipid profile was not modified	(79)
	1 mg/100 g bw, orally	Cholesterol-fed rats	↑lipoprotein lipase activity ↓TG and TC levels ↑ HMG CoA reductase activity	(80)
	Aqueous extract	Cholesterol-fed rats	↓TC	(83)
	Peels	High fat-diet in rats	↓TC, LDL-C, HDL-C, and VLDL-C levels	(84)
	Leaves, 100, 200, and 300 mg	Rats, alloxan	↓TC, LDL-C, HDL-C, and VLDL-C levels	(48)
	Core	Hypercholesterolemic guinea pigs	↓TC, LDL-C, and HDL-C levels	(85)
	Fruit juice/day, for 2 weeks	Hypercholesterolemic rabbits	↓TC, LDL-C, and HDL-C levels	(86)
Anti-obesity	Flour	Human(97 women), a RCT	↓Oxidative stress ↓Body fat ↓Waist circumference	(36)
	Methanolic extracts	*In vitro*	↓Pancreatic lipase activity	(101)

ACE: angiotensin-converting enzyme; Apo B: apolipoprotein B; BG: blood glucose; BP: blood pressure; HDL-C: high- density lipoprotein cholesterol; HMG CoA: 3-hydroxy-3-methylglutaryl-coenzyme A; LDL-C: low-density lipoprotein cholesterol; RCT: randomized clinical trial; TC: total cholesterol; TG: triglyceride

## Conclusion

Metabolic syndrome is characterized by the co-occurrence of multiple changes in high blood glucose, hypertension, hyperlipidemia, and obesity, correlated with an enhanced risk of developing cardiovascular diseases and mortality. Eggplant (*S.* *melongena*) can be useful in the treatment of MetS and its complications due to possessing delphinidin (an anthocyanin), and chlorogenic acid (a phenolic acid). There have been several studies reporting that eggplant has antidiabetic properties because of its anti-oxidant effects and lowering the absorption of glucose in the digestive organs via inhibiting α-glucosidase and α-amylase activity.

On the other hand, eggplant contains abundant ACh and can act as an antihypertensive agent. Also, eggplant inhibits ACE activity and thereby can reduce high blood pressure. Clinical and animal studies have documented that eggplant has little effect on the lipid profile. Nevertheless, its inhibitory effect on pancreatic lipase activity and anti-obesity effect of eggplant have been shown. 

Generally, eggplant has shown protective effects on MetS and its complications. However, further studies on the most representative compounds of eggplant are necessary to deeply understand the mechanisms of action of this plant.
